# Supplementary material for: CGRclust: Chaos Game Representation for twin contrastive clustering of unlabelled DNA sequences
Source: BMC Genomics. 2024 Dec 18;25:1214. doi: 10.1186/s12864-024-11135-y (PMC11657719; doi:10.1186/s12864-024-11135-y)
Supplement: Supplementary file 1 — Supplementary Material 1. [file 12864_2024_11135_MOESM1_ESM.pdf]

Supplementary Material 1
Chaos Game Representation

Fatemeh Alipour[1] and Kathleen A. Hill[2], Lila Kari[1]

[1] School of Computer Science, University of Waterloo, Waterloo, ON, Canada
[2] Department of Biology, University of Western Ontario, London, ON, Canada
`falipour@uwaterloo.ca`

Introduced by Barnsley in 1988, the *Chaos Game* algorithm generates fractals by iteratively plotting points within a polygon starting from a randomly chosen initial position [2]. Its extension, *Chaos Game Representation (CGR)*, proposed by Jeffery in 1990, is an innovative method of visualizing biological sequences (e.g., DNA/RNA) by mapping them into a fractal space [6]. CGR represents the sequence data in a compact, visual format that displays the arrangement patterns of the nucleotides. As illustrated in Figure S1.1a, a *CGR square* $\mathcal{G}$, is a square with vertices (corners) in the set $V = \{(1,1),(1,0),(0,0),(0,1)\}$. The corners of the CGR square are labelled as follows: the bottom left corner is labelled by A, the top left corner is labelled by C, the top right corner is labelled by G, and the bottom right corner labelled by T. Formally, the labelling function $l : \varDelta \rightarrow V$ is defined as $l(A) = (0,0)$, $l(C) = (0,1)$, $l(G) = (1,1)$ and $l(T) = (1,0)$, where $\varDelta$ is the set $\{A, C, G, T\}$ corresponding to the four different nucleotides. Formally, the labelling function $l : \varDelta \rightarrow V$ is defined as $l(A) = (-1,-1)$, $l(C) = (-1,1)$, $l(G) = (1,1)$ and $l(T) = (1,-1)$.

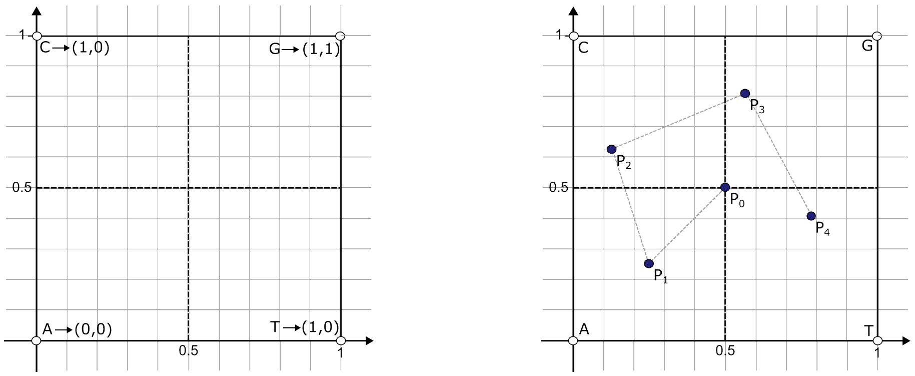

(a) (b)

Fig. S1.1: **(a)**: The CGR square $\mathcal{G}$. The vertices indicate the labelling of the corners of the CGR square. **(b)**: The CGR image X_{ACGT}, of the sequence $s = ACGT$, consists of the points p_0, p_1, p_2, p_3, p_4 illustrated in the figure.

Definition S1.1. *Let $s = a_1a_2 \dots a_n$, where $a_i \in \Delta$ for all $1 \leq i \leq n$, be a DNA sequence of length n. A CGR representation X_s of the sequence s is the set of points $X_s = \{p_0, p_1, .., p_n\} \in \mathbb{R}^2$, situated inside the CGR square, whose coordinates are defined recursively by $p_0 = (x_0, y_0) = (0, 0)$ and $p_i = \frac{p_{i-1}+l(a_i)}{2}$, for all $1 \leq i \leq n$.*

The plotting begins from the center of the CGR square. The first nucleotide in the sequence is plotted halfway between the center and the vertex corresponding to that nucleotide. Each subsequent nucleotide (from the 5' to the 3' end) is plotted halfway between the previous point and the vertex representing the current nucleotide. This iterative process generates a pattern of points within the square, reflecting the order and frequency of nucleotides in the DNA sequence. For example if $s = ATGC$, then $X_s = \{p_0, p_1, p_2, p_3, p_4\}$ is the set of points shown in Figure S1.1b. The points plotted in the CGR represent nucleotide occurrences in the sequence and the last plotted point of a CGR could in theory (at infinite resolution) be used to recover the original DNA sequence [5, 4, 1].

Frequency CGR (FCGR) is a quantified variant of CGR: An *FCGR of resolution k* is a $2^k \times 2^k$ grayscale image wherein the intensity of each pixel directly corresponds to the frequency of its corresponding k-mer. FCGR is a compressed representation of DNA sequences with the compression degree indicated by the resolution k. It is obtained by dividing the CGR image into smaller, equally-sized squares and counting the number of points (or the frequency) within each square. Since FCGR smoothes the data in a grayscale image, rather than plotting each point individually, it is inherently less noisy than the traditional CGR. Similar to CGR, FCGR can identify over- and under-representation of patterns (specific arrangements or sequences of nucleotides) in DNA sequences and, thereby, can be used to determine the degree of identity between the DNA sequences of different species [3].

Definition S1.2. *Formally, a Frequency Chaos Game Representation ($FCGR_k$) of a sequence $s \in \Delta^n$ with resolution k with $n \geq k$, is a matrix in $\mathbb{R}^{2^k \times 2^k}$ derived from X_s, the CGR image of s. Its (i, j)th entry f_{ij} satisfies:*

$$f_{ij} = \frac{\textit{Number of points of } X_s \textit{ in cell } (i, j)}{n} \tag{1}$$

where cell (i, j) is the (i, j)th subsquare, starting from the bottom left, of the square $\mathcal{G}$ if we subdivide $\mathcal{G}$ into $2^k \times 2^k$ equal size subsquares.

It is worth remarking that each of the $2^k \times 2^k$ cell(i, j) corresponds to one of the 4^k k-mer, that is, the frequency f_{ij} that pixels of CGR image X_s of S falling into cell (i, j) is the frequency that the corresponding k-mer occurs in the sequence s. This numeric representation allows $FCGR_k$ to serve as a raw matrix in genomic sequence comparisons, extending its utility beyond visual applications. Note that cell (i, j) is uniquely determined by its upper left corner $(x_i, y_j) = (\frac{2(i-1)}{2^k} - 1, \frac{2j}{2^k} - 1)$, or equivalently, $i = 1 + 2^{k-1}(x_i + 1), j = 2^{k-1}(y_i + 1)$.

And the upper left (x_i, y_j) is determined by the k-mer s_k recursively as follows: $C(s_k) = (x_i, y_j)$, $C(s_k(1:k-1)) = (x'_i, y'_j)$, $C(\text{empty}) = (-1, 1)$,

$$C(s_k) = \begin{cases} ((x'_i - 1)/2, (y'_j - 1)/2), & \text{if } s(k) = A, \\ ((x'_i + 1)/2, (y'_j - 1)/2), & \text{if } s(k) = T, \\ ((x'_i + 1)/2, (y'_j + 1)/2), & \text{if } s(k) = G, \\ ((x'_i - 1)/2, (y'_j + 1)/2), & \text{if } s(k) = C. \end{cases}$$

The FCGR matrix provides a compact, informative, and versatile representation of the sequence s, suitable for both visual and numerical genomic analyses.

References

[1] Jonas S Almeida et al. "Analysis of genomic sequences by chaos game representation". In: *Bioinformatics* 17.5 (2001), pp. 429–437. DOI: 10.1093/bioinformatics/17.5.429.

[2] Michael F Barnsley. *Fractals Everywhere: New Edition*. New York: Academic Press, 1988.

[3] Pradeep Kumar Burma et al. "Genome analysis: a new approach for visualization of sequence organization in genomes". In: *Journal of Biosciences* 17 (1992), pp. 395–411. DOI: 10.1007/BF02720095.

[4] Neil J. Calkin, Eunice Y. S. Chan, and Robert M. Corless. *Computational Discovery on Jupyter*. Philadelphia, PA: Society for Industrial and Applied Mathematics, 2023. DOI: 10.1137/1.9781611977509.

[5] Nick Goldman. "Nucleotide, dinucleotide and trinucleotide frequencies explain patterns observed in chaos game representations of DNA sequences". In: *Nucleic Acids Research* 21.10 (1993), pp. 2487–2491. DOI: 10.1093/nar/21.10.2487.

[6] H Joel Jeffrey. "Chaos game representation of gene structure". In: *Nucleic Acids Research* 18.8 (1990), pp. 2163–2170. DOI: 10.1093/nar/18.8.2163.
