# Supplementary material for: CGRclust: Chaos Game Representation for twin contrastive clustering of unlabelled DNA sequences
Source: BMC Genomics. 2024 Dec 18;25:1214. doi: 10.1186/s12864-024-11135-y (PMC11657719; doi:10.1186/s12864-024-11135-y)
Supplement: Supplementary file 2 — Supplementary Material 2. [file 12864_2024_11135_MOESM2_ESM.pdf]

Supplementary Material 2
CGRclust Methodological Optimization

Fatemeh Alipour[1] and Kathleen A. Hill[2], Lila Kari[1]

[1] School of Computer Science, University of Waterloo, Waterloo, ON, Canada
[2] Department of Biology, University of Western Ontario, London, ON, Canada
falipour@uwaterloo.ca

1 Comparison of different data augmentation strategies

Data augmentation plays a critical role in contrastive clustering by significantly enhancing the model's ability to learn invariant representations from limited data. By adding different types of changes to the training data, this approach helps the model to focus on the key features that define each cluster, avoiding the trap of fitting too closely to random noise or unimportant details. This method is key for finding new patterns or types within genomic data. To understand the impact of data augmentation on the performance of the FCGR contrastive clustering model, we evaluated several strategies. This evaluation aimed to keep the balance between preserving the original sequence and adding enough variety to boost unsupervised learning. As mentioned in section 2.4 (DNA data augmentation), for each DNA sequence s_i, we applied two sets of transformations, t and $t^{'}$, from two different augmentation families, T and $T^{'}$. In this study, we looked into mutations ($mutate(\mu)$) and fragmentation ($frag(len)$) as our main augmentation methods. We compared these augmentations, looking at different strengths for both t and $t^{'}$, to see how they affected the clustering accuracy of Test 1 (order Cypriniformes), as shown in Table S2.1. The results highlighted the superiority of mutation as the augmentation technique, with mutation rates set to $\mu = 1\text{e}{-4}$ for weak augmentation and $\mu = 1\text{e}{-2}$ for strong augmentation, in comparison to alternative methods. As a result, $mutate(\mu = 1\text{e}{-4})$ and $mutate(\mu = 1\text{e}{-2})$ were selected as the default methods for weak and strong augmentations, respectively.

2 Effectiveness of two contrastive heads and weight parameter in training loss

In exploring the optimal setting for the weight parameter α in the training loss function $L_{train} = \alpha L_{ins} + (1 - \alpha)L_{clu}$, we tested eleven different values ranging from 0 to 1. Figure S2.1 shows the experimental results, spanning four distinct datasets in Tests 1-4 (Group 1 dataset), indicating that the value of 0.7 for α consistently delivered either highest or close to highest accuracy. Furthermore, it was observed that values within the range of 0.5 to 0.8 generally yielded superior outcomes, suggesting a robust zone of performance for α across varying data

Table S2.1: **Impact of various augmentation techniques on clustering accuracy of Test 1 (clustering of order Cypriniformes).** This assessment focuses on the effectiveness of the data augmentation technique used for weak and strong augmentation in CGRclust pipeline.

**Weak augmentation (t)**	**Strong augmentation (t')**	**Accuracy**
$mutate(\mu = 1\text{e}{-4})$	$mutate(\mu = 1\text{e}{-3})$	74.50%
**mutate(μ = 1e−4)**	**mutate(μ = 1e−2)**	**94.78%**
$mutate(\mu = 1\text{e}{-3})$	$mutate(\mu = 1\text{e}{-2})$	72.69%
$frag(l = 0.99 \times sequence\ length)$	$frag(l = 0.8 \times sequence\ length)$	83.53%
$frag(l = 0.95 \times sequence\ length)$	$frag(l = 0.8 \times sequence\ length)$	75.50%
$frag(l = 0.95 \times sequence\ length)$	$frag(l = 0.7 \times sequence\ length)$	75.50%
$frag(l = 0.95 \times sequence\ length)$	$frag(l = 0.6 \times sequence\ length)$	86.75%
$frag(l = 0.95 \times sequence\ length)$	$frag(l = 0.5 \times sequence\ length)$	72.49%
$frag(l = 0.9 \times sequence\ length)$	$frag(l = 0.8 \times sequence\ length)$	70.28%

conditions. This finding emphasizes the critical role of α in balancing the contributions of instance-level and cluster-level loss components to achieve optimal training results.

3 Optimization of temperature parameters

A thorough hyperparameter optimization for the twin deep clustering model, emphasizing the instance- and cluster-level temperature parameters (τ_I and τ_C) in ICH and CCH, was crucial for enhancing clustering accuracy. Figure S2.2 depicts a 3D visualization of this optimization, examining ten distinct values for each temperature parameter in the range $[0.1, 1]$, revealing a detailed accuracy landscape within the parameter spectrum for four datasets in Tests 1-4 (Group 1 dataset). This analysis, illustrated using four 3D surface plots, reveals that while the optimal values of these two hyperparameters vary slightly across four datasets, choosing $\tau_I = 0.1$ and $\tau_C = 1.0$ consistently yields relatively high accuracy across all datasets, comparable to the highest accuracy observed in each dataset. This detailed adjustment of parameters notably improved the clustering of mtDNA sequences over standard unsupervised methods at different taxonomic levels. This advancement aligns with the hypothesis that a lower τ_I encourages individual instance differentiation, aligning with the ICH's aim, while a higher τ_C enhances group discrimination, mirroring the CCH's objective [1].

4 Majority voting scheme

The integration of ensemble learning, through majority voting, into clustering methodologies has significantly enhanced the clustering accuracy of genomic sequences, as highlighted in [3, 2]. While majority (or hard) voting operates on the most common prediction among multiple models, soft voting takes into account

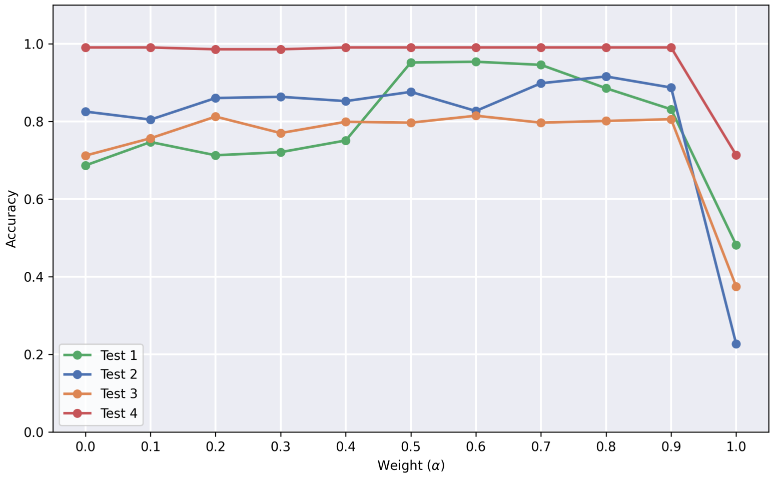

Fig. S2.1: **Experimental results illustrating the effect of the weight parameter α in the training loss function across the Group 1 dataset.** Testing of eleven values from 0 to 1 revealed that values between 0.5 and 0.8 generally delivered superior results. The default $\alpha = 0.7$ consistently yielded the highest or near-highest accuracy in Tests 1-4.

the probability distributions of outcomes, offering a consensus that often leads to higher precision. This method applied to the outcomes of the proposed twin contrastive clustering models, mitigates variance from random initialization, and leverages collective model intelligence, thereby improving the robustness and reliability of clustering outcomes. Both soft and hard majority voting, by combining each model's prediction, has proven more effective in clustering the four datasets used in this study, as depicted in Figure S2.3.

References

[1] Anna Kukleva et al. "Temperature schedules for self-supervised contrastive methods on long-tail data". In: *arXiv preprint arXiv:2303.13664* (2023). DOI: 10.48550/arXiv.2303.13664.

[2] Pablo Millán Arias, Kathleen A Hill, and Lila Kari. "*i*DeLUCS: a deep learning interactive tool for alignment-free clustering of DNA sequences". In: *Bioinformatics* 39.9 (2023), btad508. DOI: 10.1093/bioinformatics/btad508.

[3] Pablo Millán Arias et al. "DeLUCS: deep learning for unsupervised classification of DNA sequences". In: *PLOS One* 17.1 (2022), e0261531. DOI: 10.3389/fmolb.2023.1305506.

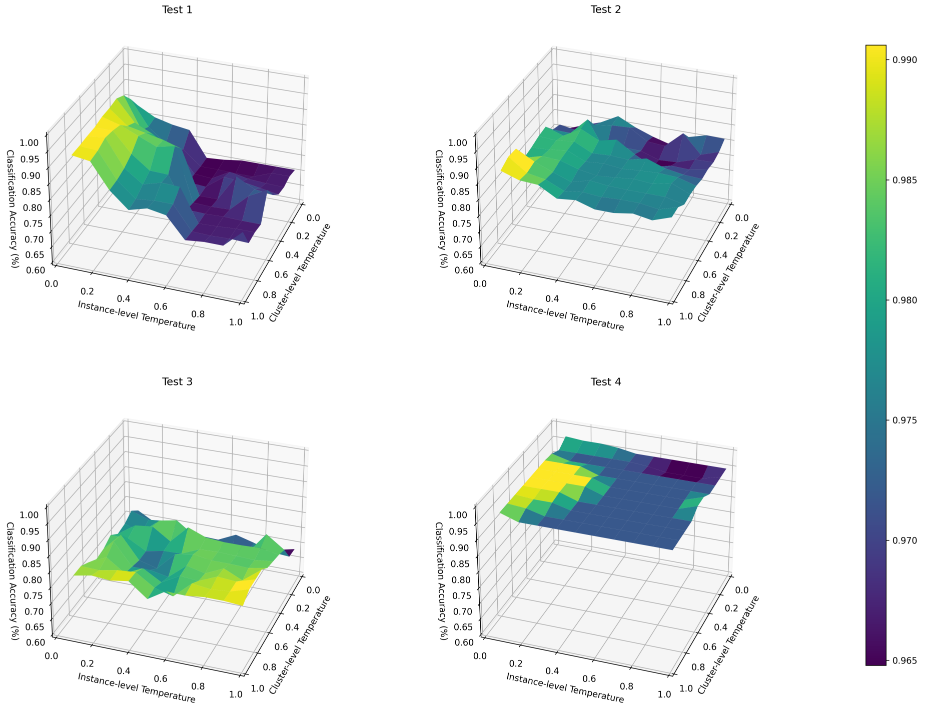

Fig. S2.2: **3D visualization of hyperparameter optimization for the twin deep clustering model in Tests 1-4, focusing on τ_I and τ_C parameters.** This figure illustrates the exhaustive search across a parameter range of [0.1, 1], presenting the accuracy landscape and emphasizing the optimal configurations for small values of τ_I (e.g. 0.1) and larger values of τ_C (e.g. 1.0), which considerably improve mitochondrial DNA sequence clustering at different taxonomic levels.

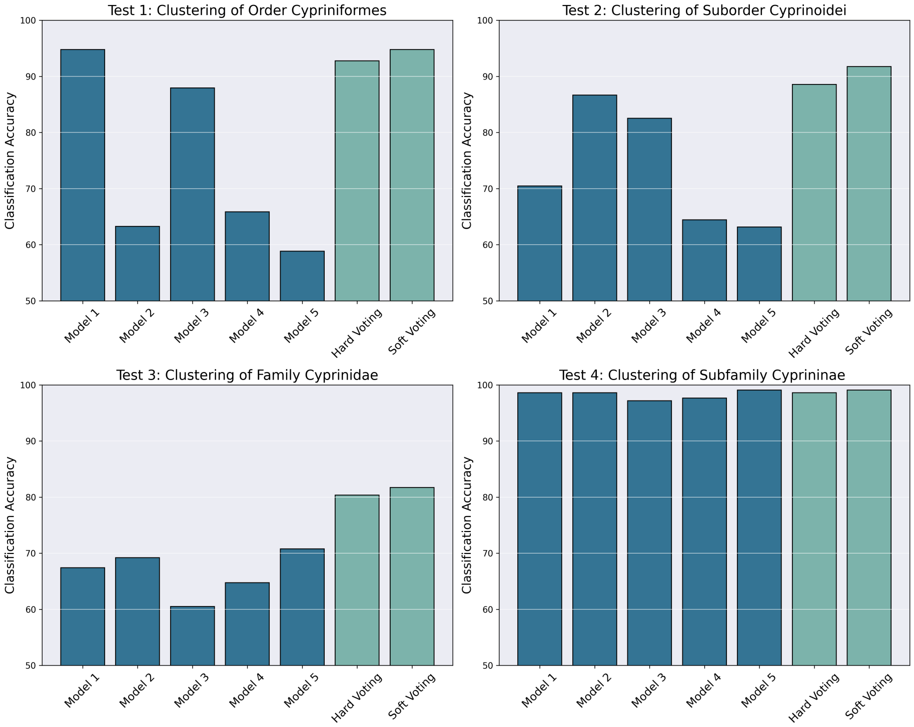

Fig. S2.3: **Comparative analysis of clustering accuracy across four datasets using hard and soft majority voting.** The bar plot illustrates the significant improvement in clustering accuracy when employing a voting scheme within the framework of twin contrastive clustering models, highlighting the enhanced accuracy in genomic sequence classification.
