# Supplementary material for: CGRclust: Chaos Game Representation for twin contrastive clustering of unlabelled DNA sequences
Source: BMC Genomics. 2024 Dec 18;25:1214. doi: 10.1186/s12864-024-11135-y (PMC11657719; doi:10.1186/s12864-024-11135-y)
Supplement: Supplementary file 3 — Supplementary Material 3. [file 12864_2024_11135_MOESM3_ESM.pdf]

Supplementary Material 3
Twin Contrastive Learning

Fatemeh Alipour[1] and Kathleen A. Hill[2], Lila Kari[1]

[1] School of Computer Science, University of Waterloo, Waterloo, ON, Canada
[2] Department of Biology, University of Western Ontario, London, ON, Canada
falipour@uwaterloo.ca

1 Twin contrastive learning

Twin Contrastive Clustering (TCL) is a machine learning method that utilizes the principles of contrastive learning to enhance clustering performance, especially in tasks involving complex or high-dimensional data, such as images, or text [3, 2]. In TCL, during the training phase, the backbone, instance-level contrastive head (ICH), and cluster-level contrastive head (CCH) undergo joint optimization based on the following twin contrastive loss function:

$$L_{train} = L_{ins} + L_{clu} \tag{1}$$

Here, L_{ins} represents the instance-level contrastive loss calculated using ICH, while L_{clu} indicates the cluster-level contrastive loss computed with CCH.

Instance-level contrastive learning strives to maximize similarities among positive pairs while minimizing those among negative pairs. Ideally, achieving clustering would involve defining within-class instance pairs as positive and between-class instance pairs as negative. However, lacking prior label information, we compromise by constructing instance pairs based on data augmentations. Specifically, positive pairs comprise samples augmented from the same DNA sequence, while negative pairs include all other sample pairs. For a batch of size N, we apply two types of data augmentations to each DNA sequence, s_i, resulting in 2N augmented samples denoted as $\tilde{s}_1, \tilde{s}_2, ..., \tilde{s}_{2i-1}, \tilde{s}_{2i}, ...\tilde{s}_{2N}$. Each original sample s_i generates $2N-1$ pairs with the other samples. Among these pairs, we consider the pair formed by s_i and its corresponding augmented sample as positive, while considering the remaining $2N-2$ pairs as negative.

Prior to implementing instance-level contrastive learning, the features were transformed into a subspace using a two-layer nonlinear MLP denoted as $g_I(\cdot)$. The InfoNCE loss [1] is utilized to optimize pairwise similarities, as defined by the following equation (suppose $\tilde{s}_i$ forms a positive pair with $\tilde{s}_j$):

$$l_i = -\log \frac{\exp(sim(z_i, z_j)/\tau_I)}{\sum_{k=1}^{2N} 1_{k\neq i} \exp sim(z_i, z_k)/\tau_I} \tag{2}$$

where $z_i = g_I(X_{s_i})$, sim is pair-wise similarity measured using cosine distance, and τ_I is the instance-level temperature parameter to control the smoothness of

the probability distribution. The instance-level contrastive loss is an average of the above loss over the $2N$ samples in the batch:

$$L_{ins} = \frac{1}{2N} \sum_{i=1}^{2N} l_i \tag{3}$$

When a sample is mapped into a subspace with dimensions equal to the number of clusters, the i-th component of its feature denotes the likelihood of it belonging to the i-th cluster. If the desired number of target clusters is denoted as C, similar to the instance-level contrastive head, we employ another two-layer MLP $g_C(\cdot)$ to transform the features into a space of dimensionality C. Let $Y = \{y_1, y_3, ..., y_{2N-1}\} \in \mathbb{R}^{N \times C}$ be the cluster assignment probabilities of the batch (of size $2N$) under the weak augmentation T , and $Y^{'} = \{y_2, y_4, ..., y_{2N}\} \in \mathbb{R}^{N \times C}$ be the cluster assignment probabilities under the strong augmentation $T^{'}$, where $y_i = g_C(X_{s_i})$. The columns of Y and $Y^{'}$ correspond to the cluster distributions. We denote the i-th column of Y as $\hat{y}_{2i-1}$ and the i-th column of $Y^{'}$ as $\hat{y}_{2i}$. The InfoNCE loss was once again utilized to optimize pairwise similarities of the representation of cluster i under the weak (and strong) data augmentation, as defined by the following equation:

$$\hat{l}_i = -\log \frac{\exp(sim(\hat{y}_i, \hat{y}_j))/\tau_C}{\sum_{k=1}^{2C} 1_{k \neq i} \exp sim(\hat{y}_i, \hat{y}_k)/\tau_C} \tag{4}$$

where sim is pair-wise similarity measured using cosine distance, and τ_C is the cluster-level temperature parameter to control the smoothness of the probability distribution. To achieve more balanced clustering outcomes we added cluster entropy to the average of the above cluster-level loss over the C clusters to define the cluster-level contrastive loss:

$$L_{clu} = \frac{1}{2C} \sum_{i=1}^{2C} \hat{l}_i + \sum_{i}^{2C} (P(\hat{y}_i) \log P(\hat{y}_i)) \tag{5}$$

Here, $P(\hat{y}_{2i-1})$ and $P(\hat{y}_{2i})$ represent the probability of assigning cluster i under weak and strong augmentation, respectively.

References

[1] Ting Chen et al. “A Simple Framework for Contrastive Learning of Visual Representations”. In: *Proceedings of the 37th International Conference on Machine Learning*. Ed. by Hal Daumé III and Aarti Singh. Vol. 119. Proceedings of Machine Learning Research. San Diego, CA: PMLR, 13–18 Jul 2020, pp. 1597–1607. URL: `https://proceedings.mlr.press/v119/chen20j.html`.

[2] Yunfan Li et al. “Contrastive clustering”. In: *Proceedings of the AAAI Conference on Artificial Intelligence*. Vol. 35. 10. 2021, pp. 8547–8555. DOI: `10.1609/aaai.v35i10.17037`.

[3] Li Yunfan et al. "Twin Contrastive Learning for Online Clustering". In: *International Journal of Computer Vision* 130 (2022), pp. 2205–2221. DOI: 10.1007/s11263-022-01639-z.
