# Supplementary material for: CGRclust: Chaos Game Representation for twin contrastive clustering of unlabelled DNA sequences
Source: BMC Genomics. 2024 Dec 18;25:1214. doi: 10.1186/s12864-024-11135-y (PMC11657719; doi:10.1186/s12864-024-11135-y)
Supplement: Supplementary file 4 — Supplementary Material 4. [file 12864_2024_11135_MOESM4_ESM.pdf]

Supplementary Material 4
Optimal Threshold Values Across Datasets for MeShClust v3.0

Fatemeh Alipour[1] and Kathleen A. Hill[2], Lila Kari[1]

[1] School of Computer Science, University of Waterloo, Waterloo, ON, Canada
[2] Department of Biology, University of Western Ontario, London, ON, Canada
falipour@uwaterloo.ca

MeShClust v3.0 [1] is a density-based clustering tool that does not allow for the pre-definition of cluster numbers. Instead, it utilizes a threshold parameter that defines the identity score needed for sequences to be grouped into the same cluster. This identity score, which typically ranges from 0 to 1, indicates the degree of similarity between sequences. Adjusting this threshold is crucial as it influences the clustering granularity: higher thresholds group only very similar sequences, while lower thresholds accommodate greater dissimilarity within clusters. This flexibility is particularly beneficial in genomic studies, allowing MeShClust v3.0 to tailor clustering to varying levels of sequence similarity across different datasets or taxonomic levels.

To address potential discrepancies between expected and actual cluster counts caused by automatic threshold selection, we manually investigated various identity score thresholds within the $[0, 1]$ range for each dataset. This approach enabled the selection of an optimal threshold for each clustering test, aligning with the desired cluster count. The optimal threshold values for each of the thirteen datasets tested are detailed in Table S4.1. As shown in the first four rows of Table S4.1, progressing to lower taxonomic levels where sequences become more similar necessitated increasing the identity score threshold to achieve the correct number of clusters.

Table S4.1: Optimal threshold values for identity scores using MeShClust v3.0 across thirteen real datasets. The identity threshold is a parameter that specifies the minimum similarity score required for sequences to be grouped together in a cluster.

Test	Dataset	Threshold
1	Cypriniformes	0.670
2	Cyprinoidei	0.695
3	Cyprinidae	0.814
4	Cyprininae	0.850
5	Astroviridae-unbalanced	0.437
6	Astroviridae-balanced	0.400
7	Dengue	0.750
8	HCV	0.750
9	HIV-1	0.824
10	Insecta	0.600
11	Protista	0.350
12	Fungi-phylum	0.300
13	Fungi-subphylum	0.285

References

[1] Hani Z Girgis. "MeShClust v3. 0: high-quality clustering of DNA sequences using the mean shift algorithm and alignment-free identity scores". In: *BMC Genomics* 23.1 (2022), p. 423. DOI: 10.1186/s12864-022-08619-0.
