# Supplementary material for: CGRclust: Chaos Game Representation for twin contrastive clustering of unlabelled DNA sequences
Source: BMC Genomics. 2024 Dec 18;25:1214. doi: 10.1186/s12864-024-11135-y (PMC11657719; doi:10.1186/s12864-024-11135-y)
Supplement: Supplementary file 5 — Supplementary Material 5. [file 12864_2024_11135_MOESM5_ESM.pdf]

Supplementary Material 5
CGRclust Comparative Clustering Accuracies

Fatemeh Alipour[1] and Kathleen A. Hill[2], Lila Kari[1]

[1] School of Computer Science, University of Waterloo, Waterloo, ON, Canada
[2] Department of Biology, University of Western Ontario, London, ON, Canada
falipour@uwaterloo.ca

1 Comparative Accuracies across Diverse Datasets

Figure S5.1 compares clustering accuracies for all dataset groups described in Table 1 and 2 of the manuscript. See Table S5.1 for the confidence intervals of CGRclust clustering accuracies.

2 Confidence Interval for CGRclust Clustering Accuracies

Machine learning models are often evaluated with restrictions such as limited data availability, violations of independence assumptions, and sampling biases [4, 5]. A model's confidence interval provides useful insight into the uncertainty surrounding its reported accuracy and performance metrics. The accuracy with confidence interval serves as an estimate of the model's performance on unseen data, not just on the data that the model was trained on. Typically, a 95% confidence interval is used to calculate this estimate, which provides a statistical range believed to contain the true generalized accuracy.

Under the normal approximation, the confidence interval for the clustering accuracy can be calculated from a single training-test split using the following formula:

$$CI = ACC \pm z \cdot \sqrt{\frac{ACC(1 - ACC)}{n}}$$

where ACC is the observed accuracy from the test set and n is the number of samples in the test set. For a typical confidence interval of 95%, we have z = 1.96 [4].

In deep learning, where models (e.g. CGRclust) must be trained over extended periods of time and can be computationally expensive, this method of calculating the confidence interval is especially beneficial. Table S5.1 details the clustering accuracies of twenty-five clustering tests in CGRclust with confidence intervals.

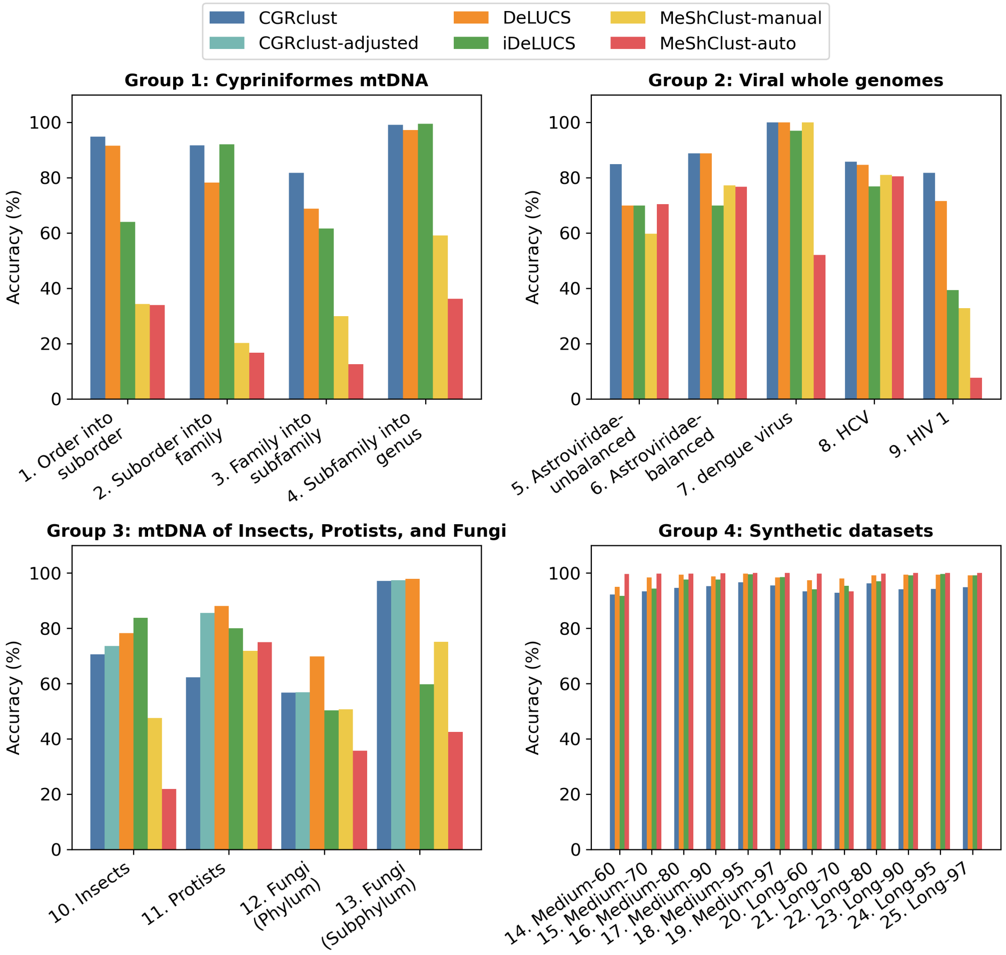

Fig. S5.1: **Comparative performance of CGRclust with other methods across four dataset groups.** This figure provides a comparison of CGRclust's clustering accuracy against DeLUCS [3], iDeLUCS [2], and MeShClust v3.0 [1]. Four dataset groups as detailed in Tables 1 and 2 of the manuscript are: Cypriniformes full mitochondrial genomes (Group 1); viral whole genomes (Group 2); mtDNA of Insects, Protists, and Fungi (Group 3); and MeShClust v3.0 synthetic datasets (Group 4). The test numbers corresponding to each dataset are represented on the x-axis of the plots. In dataset Group 3, CGRClust performance is reported with and without an adjusted α hyperparameter. The performance of MeShClust v3.0 is shown with both manual and automatic selection of identity score thresholds, with the exception of dataset Group 4.

Table S5.1: CGRclust clustering accuracies with confidence intervals.

**Test**	**Dataset**	**Number of Sequences**	**Clustering Accuracy**
1	Cypriniformes	498	94.78 ± 1.95
2	Cyprinoidei	630	91.75 ± 2.15
3	Cyprinidae	448	81.70 ± 3.58
4	Cyprininae	213	99.06 ± 1.30
5	Astroviridae-unbalanced	1089	84.94 ± 2.12
6	Astroviridae-balanced	726	88.84 ± 2.29
7	Dengue	1,628	100.00 ± 0.00
8	HCV	950	85.79 ± 2.22
9	HIV-1	1300	81.77 ± 2.10
10	Insecta	4,550	73.56 ± 1.28
11	Protista	945	85.50 ± 2.24
12	Fungi-phylum	670	56.87 ± 3.75
13	Fungi-subphylum	1,070	97.10 ± 0.96
14	Medium-60	18,210	92.26 ± 0.39
15	Medium-70	18,731	93.39 ± 0.36
16	Medium-80	20,939	94.61 ± 0.31
17	Medium-90	21,266	95.23 ± 0.29
18	Medium-95	24,039	96.57 ± 0.23
19	Medium-97	20,772	95.51 ± 0.28
20	Long-60	20,885	93.31 ± 0.34
21	Long-70	18,558	92.82 ± 0.37
22	Long-80	20,525	96.29 ± 0.26
23	Long-90	22,518	94.08 ± 0.30
24	Long-95	20,222	94.20 ± 0.32
25	Long-97	19,960	94.83 ± 0.31

References

[1] Hani Z Girgis. "MeShClust v3. 0: high-quality clustering of DNA sequences using the mean shift algorithm and alignment-free identity scores". In: *BMC Genomics* 23.1 (2022), p. 423. DOI: 10.1186/s12864-022-08619-0.

[2] Pablo Millán Arias, Kathleen A Hill, and Lila Kari. "*i*DeLUCS: a deep learning interactive tool for alignment-free clustering of DNA sequences". In: *Bioinformatics* 39.9 (2023), btad508. DOI: 10.1093/bioinformatics/btad508.

[3] Pablo Millán Arias et al. "DeLUCS: deep learning for unsupervised classification of DNA sequences". In: *PLOS One* 17.1 (2022), e0261531. DOI: 10.3389/fmolb.2023.1305506.

[4] Sebastian Raschka. "Model evaluation, model selection, and algorithm selection in machine learning". In: *arXiv preprint arXiv:1811.12808* (2018). DOI: 10.48550/arXiv.1811.12808.

[5] Peter Steinbach et al. "Machine Learning State-of-the-Art with Uncertainties". In: (2022). ICLR22, ML Evaluation Standards workshop. DOI: 10.48550/ARXIV.2204.05173. URL: https://ml-eval.github.io/accepted-papers/#11.
