# Supplementary material for: CGRclust: Chaos Game Representation for twin contrastive clustering of unlabelled DNA sequences
Source: BMC Genomics. 2024 Dec 18;25:1214. doi: 10.1186/s12864-024-11135-y (PMC11657719; doi:10.1186/s12864-024-11135-y)
Supplement: Supplementary file 6 — Supplementary Material 6. [file 12864_2024_11135_MOESM6_ESM.pdf]

Supplementary Material 6
CGRclust Training Times Across Different Tests

Fatemeh Alipour[1] and Kathleen A. Hill[2], Lila Kari[1]

[1] School of Computer Science, University of Waterloo, Waterloo, ON, Canada
[2] Department of Biology, University of Western Ontario, London, ON, Canada
falipour@uwaterloo.ca

The training times for CGRclust were recorded for each clustering test, as detailed in Table S6.1. As the results show, the time required for each clustering task is directly correlated with the number of genome sequences in the dataset, indicating that increased dataset size increases computational complexity and extends clustering duration—an expected behavior in clustering algorithms. The length of the DNA sequences, however, influences the training time minimally, impacting only the pre-processing phase. In the pre-processing phase, genome sequences are transformed into Frequency Chaos Game Representations (FCGRs), converting them into 64x64 matrices when $k = 6$. This transformation ensures uniformity in data handling, as all sequences, regardless of their original lengths, are represented by matrices of the same dimensions. Consequently, after this conversion to FCGRs, the clustering algorithm processes datasets of these fixed-sized matrices, making the original sequence lengths irrelevant in terms of computational load during the clustering stage. As a result of this fixed-size representation, the clustering algorithm is focused on analyzing patterns within FCGRs rather than managing different sequence lengths.

Table S6.1: CGRclust training time across twenty-five clustering tests

Test	Dataset	Number of Sequences	Time (seconds)
1	Cypriniformes	498	551.240
2	Cyprinoidei	630	634.810
3	Cyprinidae	448	476.283
4	Cyprininae	213	413.320
5	Astroviridae-unbalanced	1,089	843.984
6	Astroviridae-balanced	726	712.654
7	Dengue	1,628	1146.037
8	HCV	950	720.595
9	HIV-1	1300	961.421
10	Insecta	4,550	2255.074
11	Protista	945	725.403
12	Fungi-phylum	670	662.347
13	Fungi-subphylum	1,070	859.819
14	Medium-60	18,210	7973.674
15	Medium-70	18,731	8170.432
16	Medium-80	20,939	9167.305
17	Medium-90	21,266	9275.432
18	Medium-95	24,039	8764.424
19	Medium-97	20,772	8731.778
20	Long-60	20,885	8941.603
21	Long-70	18,558	8023.114
22	Long-80	20,525	8886.094
23	Long-90	22,518	9592.560
24	Long-95	20,222	8764.240
25	Long-97	19,960	8731.778
