# Supplementary material for: CGRclust: Chaos Game Representation for twin contrastive clustering of unlabelled DNA sequences
Source: BMC Genomics. 2024 Dec 18;25:1214. doi: 10.1186/s12864-024-11135-y (PMC11657719; doi:10.1186/s12864-024-11135-y)
Supplement: Supplementary file 7 — Supplementary Material 7. [file 12864_2024_11135_MOESM7_ESM.pdf]

Supplementary Material 7
Comparative Evaluation of Phylonium

Fatemeh Alipour[1] and Kathleen A. Hill[2], Lila Kari[1]

[1] School of Computer Science, University of Waterloo, Waterloo, ON, Canada
[2] Department of Biology, University of Western Ontario, London, ON, Canada
falipour@uwaterloo.ca

Phylonium is a program created to quickly estimate evolutionary distances between closely related genomes, useful for applications such as tracking disease outbreaks using whole-genome sequencing. It achieves speed by indexing a single reference sequence and piling all other sequences onto it, creating an approximate multiple-sequence alignment from which the distances are calculated. Phylonium is particularly useful in genomic epidemiology, where rapid and accurate distance estimations are crucial [1].

To compare CGRClust with phylonium, we also tested phylonium with all twenty-five datasets used in the CGRclust study. Phylonium was very fast, generating the evolutionary distance matrix in under a minute for datasets in Groups 1 and 2. As the selection of an appropriate reference can significantly affect the accuracy of phylonium, and Given the variety in our datasets and the complexity of choosing a reference for each, we utilized phylonium's –2pass option. This option allows phylonium to select a central sequence as the reference after an initial run, which generally works well.

After generating the evolutionary distance matrix, we employed Ward's method to construct phylogenetic trees, minimizing the variance within each cluster. We then cut each tree at heights corresponding to the expected number of clusters. Table S7.1 presents the clustering accuracies of CGRclust compared to phylonium for the five datasets, out of twenty-five, where phylonium successfully generated distance matrices.

Table S7.1: Comparison of clustering accuracies between CGRclust and phylonium for five out of twenty-five datasets where phylonium successfully generated distance matrices.

Test	Dataset	CGRclust Accuracy	Phylonium Accuracy
1	Cypriniformes (Order to Suborder)	94.78%	90.76%
2	Cypriniformes (Suborder to Family)	91.75%	71.75%
3	Cypriniformes (Family to Subfamily)	81.70%	72.77%
4	Cypriniformes (Subfamily to Genus)	99.06%	99.53%
9	Human Immunodeficiency Virus 1	81.77%	64.15%

For the remaining 20 tests, phylonium generated matrices with NaN values, preventing us from generating trees and conducting clustering. These datasets

included more heterogeneous sequences, demonstrating a limitation of phylonium, namely that it is primarily effective for closely related samples. As noted by Klötzl et al. [1], “when phylonium is applied to divergent sequences: it cannot find any anchors and hence cannot estimate the distance. This restriction to closely related sequences makes phylonium suitable for applications like genomic epidemiology, but not as a general tool for estimating phylogenetic distances.”

References

[1] Fabian Klötzl and Bernhard Haubold. “Phylonium: Fast estimation of evolutionary distances from large samples of similar genomes”. In: *Bioinformatics* 36.7 (2020), pp. 2040–2046.
